# NFU-Enabled FASTA: moving bioinformatics applications onto wide area networks

**DOI:** 10.1186/1751-0473-2-8

**Published:** 2007-11-26

**Authors:** Erich J Baker, Guan N Lin, Huadong Liu, Ravi Kosuri

**Affiliations:** 1 Department of Computer Science, School of Engineering and Computer Science, Baylor University, Waco, TX, USA; 2 Department of Computer Science, University of Missouri, Columbia, MO, USA; 3 Department of Computer Science, University of Tennessee, Knoxville, TN, USA

## Abstract

**Background:**

Advances in Internet technologies have allowed life science researchers to reach beyond the lab-centric research paradigm to create distributed collaborations. Of the existing technologies that support distributed collaborations, there are currently none that simultaneously support data storage and computation as a shared network resource, enabling computational burden to be wholly removed from participating clients. Software using computation-enable logistical networking components of the Internet Backplane Protocol provides a suitable means to accomplish these tasks. Here, we demonstrate software that enables this approach by distributing both the FASTA algorithm and appropriate data sets within the framework of a wide area network.

**Results:**

For large datasets, computation-enabled logistical networks provide a significant reduction in FASTA algorithm running time over local and non-distributed logistical networking frameworks. We also find that genome-scale sizes of the stored data are easily adaptable to logistical networks.

**Conclusion:**

Network function unit-enabled Internet Backplane Protocol effectively distributes FASTA algorithm computation over large data sets stored within the scaleable network. In situations where computation is subject to parallel solution over very large data sets, this approach provides a means to allow distributed collaborators access to a shared storage resource capable of storing the large volumes of data equated with modern life science. In addition, it provides a computation framework that removes the burden of computation from the client and places it within the network.

## Background

Internet technologies have allowed life science researchers to reach beyond the lab-centric paradigm to create distributed collaborations. There have recently been several examples of successful geographically disparate research projects that strive to leverage research expertise, data and analysis from different locations [1-3]. In each instance, there is a distinction between collaborative data storage, access, curation, and the distribution of computation resources. Technology limitations tend to produce systems that rely on centralized data storage resources with a mixture of client or server-side computation, straining the effectiveness of these models as the volume of data or computation complexity exceeds bandwidth, physical storage or computation capacity. While there is as yet no clear technology that satisfies both distributed data storage and computation simultaneously, there are distinct approaches. Typical metaphors for distributed collaboration include federated databases, GRID and Peer-to-Peer(P2P)-based data computation and storage, semantic networks, and strategies that attempt to combine these concepts. For example, semantic networks provide interesting solutions for data analysis and maintaining data integrity but do not offer solutions for computation [4,5]. GRID systems provide reasonable approaches to solve data storage and computation but are not acceptable for every scenario because their highly structured nature requires GRID clients to maintain independent operational integrity, tightly coupled processors, and susceptibility to malicious attacks [6,7]. Semantic GRIDs and P2P networks are attempts to alleviate these issues and have had variable success [8,9].

To address issues of distributed storage, recent efforts have integrated networking and storage by providing storage to the end user as a shared resource of the network, analogous to the way the current Internet provides bandwidth as a shared resource. This process, defined as Logistical Networking [10], describes a storage infrastructure created by employing a generic best-effort service for storage. Stronger services are provided as the higher layers of the network storage stack in accordance with end-to-end design principles, including traffic-proportional burdens on network services [11]. The specific implementation of this model as described herein, called the Internet Backplane Protocol (IBP), has created a test bed offering access through the Internet to greater than 35 terabytes of storage space, on over 250 locally maintained storage depots spread across 20 countries [12,13].

The abstracted layers comprising IBP services have been well described [10,13,14]. Briefly, it is a middleware for managing and using remote storage while simultaneously allowing users access to standard Internet resources. Here, we focus on a particular extension called the Network Function Unit (NFU), a generic, best effort end-to-end approach to provide computation-enabled IBP nodes for data storage and transformation [15]. NFU operations are grouped libraries, enabling their hierarchal management, and bounded by duration of execution. Operations are *static *or *dynamic*, and utilized as IBP node built-in modules or user-submit executions, respectively [11]. In this paper, we describe a practical bioinformatics and life science software application using NFU-enabled IBP as a means of both data storage and computation, filling a much-needed gap in research conducted as part of distributed collaborations.

The model system presented here uses a modified form of the FASTA algorithm that distributes computation and storage resources across nodes in an IBP network. The FASTA suite of tools was chosen because it is a widely distributed biologically-relevant set of algorithms used to produce sequence alignments in large search space and has been shown to be amenable to parallel computation [16]. The basic algorithm relies on local sequence alignment to find similarity, scores possible results using a largely heuristic engine and completes the possible solution sets using a modified Smith-Waterman algorithm [17]. By using FASTA we demonstrate that in cases where parallel computation is possible, NFU-enabled IBP provides a powerful option for both data storage and computation across wide area networks.

## Implementation

### System Architecture

**Figure 1:**
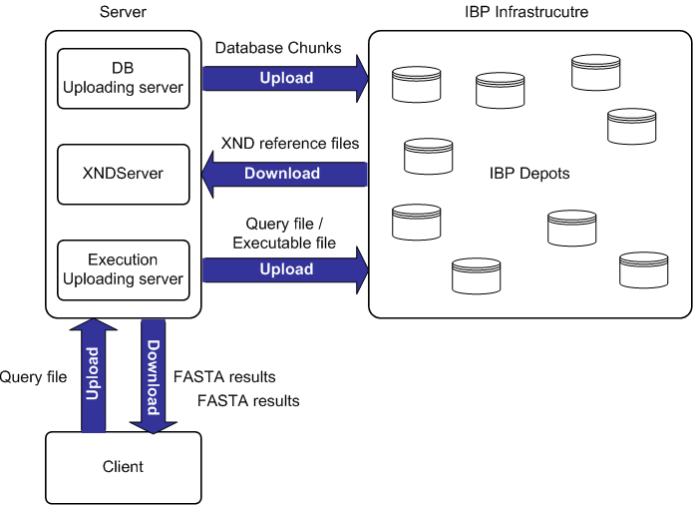
**High level schema of NFU-enabled FASTA**. The burden of database maintenance and distribution within the IBP network is handled by the server using the LoRS upload tool and associated XND files to catalog distributed database location and replicate. Following a request for execution, the server retrieves the query file from the client and uploads the query file and modified FASTA executable onto NFU-enabled storage depots where the appropriate database chunks reside. The FASTA results are download directly from the network, modified if necessary, and returned to the client.

**Figure 2:**
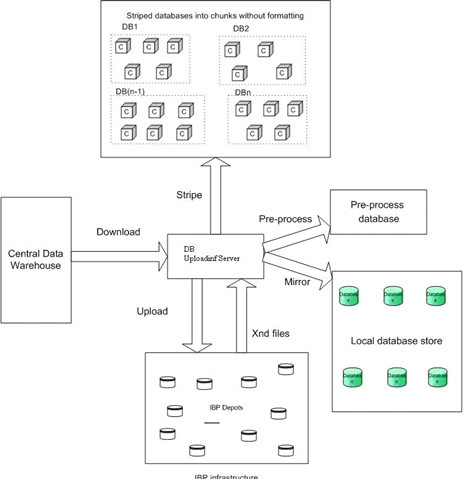
**High level schema for DB uploading server**. The DB uploading server component, residing within the local IBP server, downloads the appropriate sequence complement from a centralized data warehouse (e.g., the FTP site at NCBI). It preprocesses chunks to ensure proper formatting, stripes the databases and uploads them into the IBP network. It also maintains a local mirror of the latest copy downloaded from the central warehouse; the backup store may be used if required depots are unavailable.

**Figure 3:**
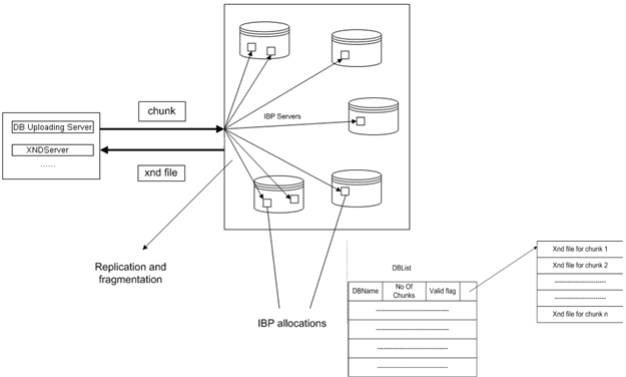
**Chunk upload and XND server**. (A) The DB Uploading Server uses the LoRS upload tool to upload each chunk of a database. The chunk is replicated and fragmented depending on the parameters given to the upload tool before being stored in the IBP network as IBP allocations. (B) The DBList maintains a list of all the databases that have been uploaded and are available, and the information associated with them (e.g., the no of chunks, the list of xnd files, etc).

**Figure 4:**
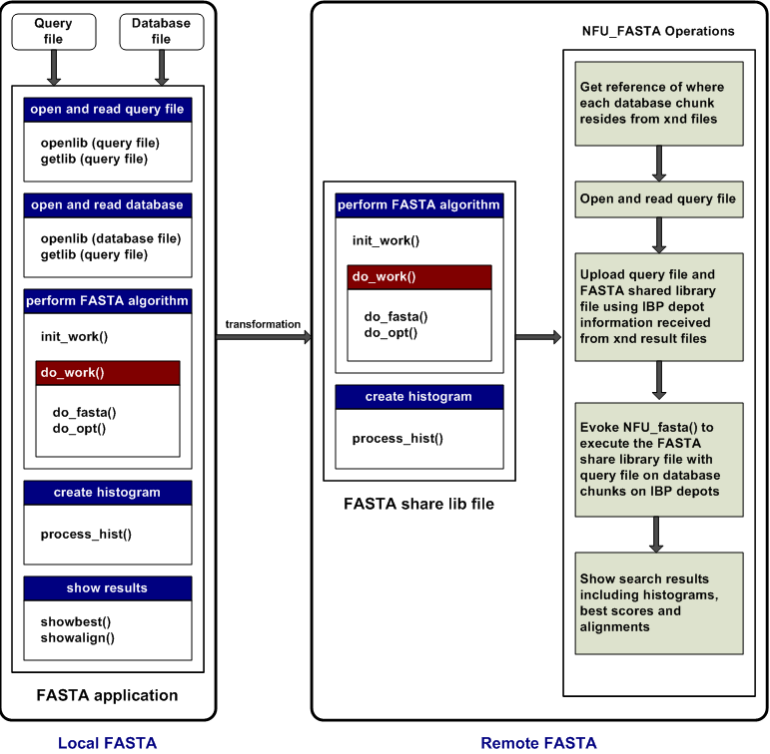
**High level schema for execution uploading server**. The Execution Uploading Server component in the Server transforms local FASTA into remote FASTA application. Briefly, a shared FASTA library file is created by stripping out the FASTA algorithm and histogram creation portions from the FASTA package and converting them to a static shared library. Since the NFU computation service enabled on the IBP depots is implemented in the C programming language, the transformation of FASTA code to shared library files is also implemented in C.

The overall server architecture consists of a DB Uploading Server, XNDServer, and Execution Uploading Server; a high-level schema is described in Figure 1. The DB Uploading Server and XNDServer are adapted from the IBP-BLAST system as previously reported [18]. Briefly, the DB Uploading Server partitions the original FASTA-formatted databases into smaller 'chunks,' which are uploaded into the logistical network through the LoRs upload tool (Figures 2, 3). This operation returns XND files (xml-formatted reference files), indexed references to uploaded files which are managed by the XNDServer (Figure 3). The Execution Uploading Server obtains the database chunk network location reference from the XNDServer and uploads the query file and FASTA executable file to the locations where the data resides for FASTA execution (Figure 4). Results of all individual chunk executions are returned to the server by the depot where they are merged to produce complete results for each query. Ultimately, these are downloaded by client side services to be displayed to the user.

### FASTA Shared Library File

The creation of NFU-compatible FASTA algorithm and histogram code was accomplished by stripping this code from the original FASTA algorithm and converting it into a shared static library (Figure 4). It was then implemented in the C programming language for NFU compatibility. An interface, called NFU_FASTA, acts as a façade between the FASTA shared library and NFU functions; it converts FASTA library function parameters into NFU function parameters which perform FASTA searches on IBP-stored biological data. Invoked IBP depots, or nodes, perform FASTA sequence analysis only on data residing within that particular depot using techniques analogous to parallel FASTA [16]. It returns results to the server through NFU_FASTA download capacities. After the result files are obtained from each queried node, the merge facility unifies the intermediate output files through a text merge that produces the final output.

### Experimental System Design

In order to test this software implementation and to ascertain the strengths of distributing both data and analysis tools over IBP logistical networks, FASTA alignment of genome-scale nucleotide data was performed under various conditions. In System 1, *Local FASTA with original database system*, the test databases were stored in a FASTA formatted form in the local directory. A script was used to take a set of accession numbers in a file as input, fetch the corresponding FASTA sequences from the NCBI [19], and align them against specified databases. A locally installed FASTA program was used for the alignment operation and various time parameters were monitored. System 2, *FASTA with local IBP network*, used a similar setup to System 1; here, test datasets were "chunked" to mimic the stripped copies stored in IBP networks. System 3 represents the *IBP-FASTA *software described in this paper. One local server was dedicated as a client server while three others participate in the IBP network. Each node in the test system contained an enabled NFU. Test datasets were chunked and distributed within the test IBP network in a similar fashion as System 2.

Four benchmark tests were performed using the design systems described. All three systems were tested in triplicate and the average times reported for (1) total response time versus query size, (2) average response time per node as a function of query size, (3) number of queries versus total response time for the *C. elegans *genome, (4) and the number of queries versus total response time for *M. musculus*. Systems 2 and 3 were tested for depot distributions of 1, 5, 10 and 20.

### Computing Resources and Data

All experiments were performed on Dell PowerEdge 1550 systems with dual Pentium 4 processors with 1 GB memory running RedHat Enterprise Linux 3.0 Workstation operating systems. The machines were designated 'earth', 'wind', 'and', 'fire,' and connected by 10/100 Mbps Ethernet to the Baylor ECS backbone. One of two FASTA-formatted nucleotide databases was used in the test system. The *Caenorhabditis elegans *genome was based on release WS162 of approximately 100 Mb [20]. The unformatted mouse chromosome 1 database was 2.3 GB and contained approximately 4 million sequences for a total of 1.8 billion nucleotides. The *M. musculus *database was obtained from the NCBI mirror site for FASTA databases [21]. Local FASTA tools were installed on all the machines [17].

## Results and Discussion

To test whether distributed collaborations could benefit from moving both bioinformatics data storage and computation onto wide area networks, we investigated whether a NFU-enabled IBP logistical networking framework could support the distribution of the FASTA algorithm over a variety of data sources. Since data storage and transformation (treated here as computation) are viewed as shared resources on the network, it was possible to create a transparent system to upload and distribute genome data and conduct similarity searches using the FASTA algorithm. As an example of the power of this approach, we tested the distribution of small (*C. elegans*) and moderate (*M. muluscus*, chromosome 1) data sets across local and remote IBP storage nodes.

**Figure 5:**
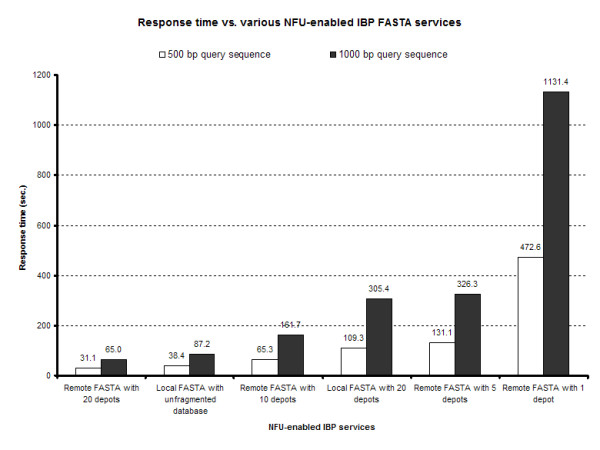
**Response time vs. NFU-enables IBP FASTA services**. FASTA-formatted genome sequence databases were either kept locally as an unformatted dataset, distributed within a local IBP node in 20 chunks, or distributed within a non-local IBP network to 1, 5, 10 or 20 nodes. The total response time versus these NFU-enabled IBP FASTA services was tested as a function of query size against the total data sets. The average of three results indicate that query sizes of 500 and 1000 bp against remote one node FASTA systems return the slowest response time. This slowdown is expected over local FASTA systems as a result of network communication times. The local FASTA system with 20 nodes has slightly better response time as compared with the system of local server with unfragmented datasets.

**Figure 6:**
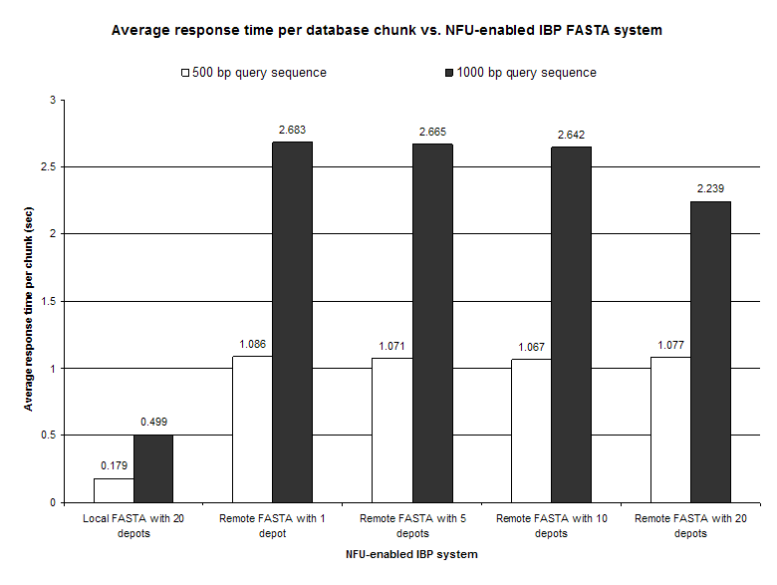
**Average response time per database chunk vs. NFU-enables IBP FASTA services**. FASTA-formatted genome sequence databases were either kept locally as an unformatted dataset, distributed within a local IBP node in 20 chunks, or distributed within a non-local IBP network to 1, 5, 10 or 20 nodes. In distributed, or chunked, systems the average response time of three efforts per node remains constant throughout the system, indicating that future speed-ups in time will be a function of the granularity of data stripping across the IBP network with a lower bound based on network communication time.

The total response time versus various NFU-enabled IBP FASTA services as a function of query size was tested against the total data sets. Results indicate that query sizes of 500 and 1000 bp against remote one node FASTA systems return the slowest response time (Figure 5). This slowdown is expected over local FASTA systems as a result of network communication times. The local FASTA system with 20 nodes had a slightly better response time as compared with the system of local server with unfragmented datasets. This indicates that there is a break-even point where server communication time balances with data stripping and replication. In distributed, or non-local, systems the average response time per node remained constant throughout the system (Figure 6), indicating that future speed-ups in time will be a function of the granularity of data stripping across the IBP network with a lower bound based on network communication time.

**Figure 7:**
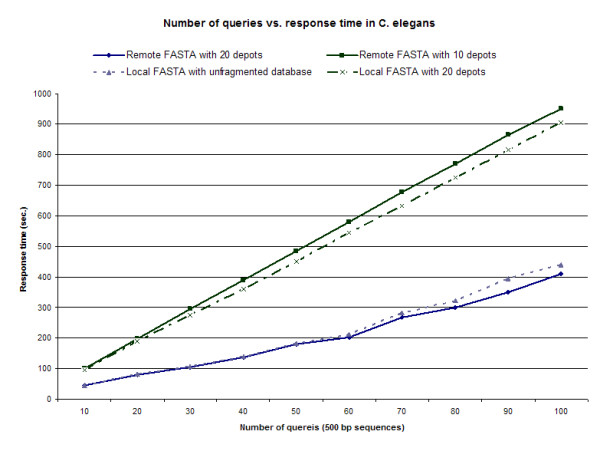
**Number of queries vs. response time in *C. elegans***. FASTA-formatted genome sequence databases were either kept locally as an unformatted dataset, distributed within a local IBP node in 20 chunks, or distributed within a non-local IBP network to 1, 5, 10 or 20 nodes. Query time of multiple 500 bp alignments against *C. elegans *databases demonstrates that as the number of distributed nodes is increased, either in local or non-local systems, there is an overall reduction in query time. Distributed data sets representing 20 nodes shows greater improvement in query time over locally run FASTA algorithms. The average of three iterations is shown.

**Figure 8:**
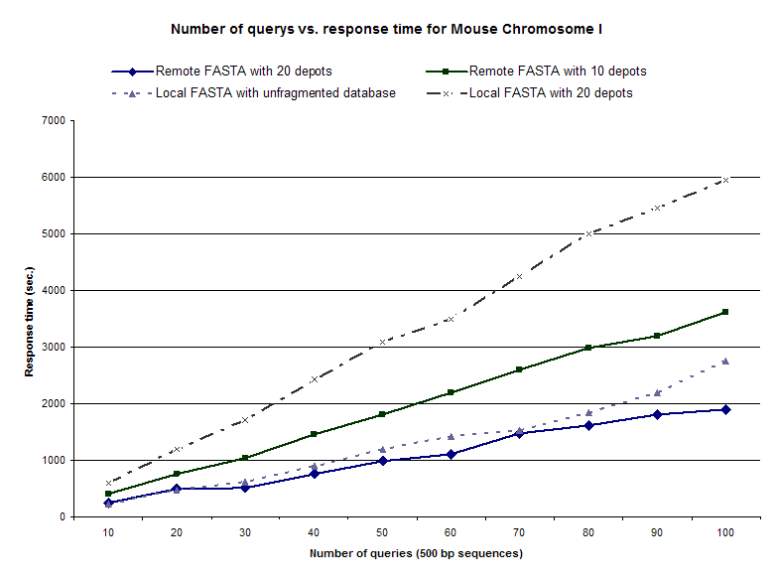
**Number of queries vs. response time in *M. musculus***. FASTA-formatted genome sequence databases were either kept locally as an unformatted dataset, distributed within a local IBP node in 20 chunks, or distributed within a non-local IBP network to 1, 5, 10 or 20 nodes. Query time of multiple 500 bp alignments against *M. musculus *databases demonstrated that as the number of distributed nodes is increased, either in local or non-local systems, there is an overall reduction in query time. Distributed data sets representing 20 nodes shows greater improvement in query time over locally run FASTA algorithms. The average of three iterations is shown.

Figures 7 and 8 demonstrate query time of multiple 500 bp alignments against *C. elegans *and *M. musculus *databases, respectively. In both cases, as the number of distributed nodes is increased, either in local or non-local systems, there is an overall reduction in query time. Distributed data sets representing 20 nodes shows greater improvement in query time over locally run FASTA algorithms. In both these scenarios the NFU-enabled nodes performed exceptionally well; the query failure rate at each node was less than 0.01%, and each query failure was identified and the query repeated on data stored on mirrored nodes. As expected, as the dataset increases in size there is a clearer benefit in the use of distributed nodes to process the algorithm. We recognize that there are insufficiencies encountered when operating parallel FASTA algorithms, as expected values depend, in part, on the size of search space which is often difficult to reconstruct accurately in stripped datasets [16]. Our results from the merged distributed-returns are not significantly different from FASTA algorithms run in a stand-alone mode (results not shown), with the vast majority of results demonstrating zero variance.

## Conclusion

As collaborative environments seek to minimize the burden of data analysis and storage with large cooperatively generated data sets there will be an increasing need to explore technology-driven storage and analysis environments. The IBP and its use of NFU-enabled nodes provides one means to reconcile these needs. Results from our preliminary tests using the FASTA algorithm as a rudimentary distributed algorithm over a network of shared datasets demonstrates the effectiveness of environments where clients may be removed from the burden of data warehousing and concurrency which hampers the research efforts of small laboratories that lack scaleable computational infrastructure. In addition, moving the burden of computation onto the network further removes the need for desktop sized machines to perform computations.

Existing solutions to collaborative data storage and analysis address restricted domains or scales, and are usually confined to tightly coupled processors. The challenge of a loosely coupled solution described here is much more daunting as the assumptions about availability and reliability of the storage and computational resources made on the local systems or grids are not valid on wide area scales. Internet solutions have to address the issues of reliability and availability of the participating nodes to deliver acceptable levels of accuracy and performance which traditionally leaves these systems vulnerable to Denial of Service (DoS) attacks and dependent on the strong semantics associated with processor-attached storage. IBP protocols have advantages over these systems because allocations can be time limited. When the lease on an allocation expires, the storage resource can be reused and all data structures associated with it can be deleted. An IBP allocation can be refused by a storage resource in response to over-allocation, much as routers can drop packets and such "admission decisions" can be based on both size and duration. Forcing time limits puts transience into storage allocation, giving it some of the fluidity of datagram delivery. More importantly, the semantics of IBP storage allocation are weaker than the typical storage service. Chosen to model storage accessed over the network, it is assumed that an IBP storage resource can be transiently unavailable. Since the user of remote storage resources is depending on so many uncontrolled remote variables, it may be necessary to assume that storage can be permanently lost. Thus, IBP is a "best effort" service. To encourage the sharing of idle resources, IBP even supports "soft" storage allocation semantics, where allocated storage can be revoked at any time. In all cases, such weak semantics mean that the level of service must be characterized statistically.

The size of bioinformatics and life science data sets makes their storage in currently available tera-scale IBP networks immediately achievable. Furthermore, the logistical networking paradigm model enables the movement of data on nodes of interest to physical proximity to clients of interest. This underscores IBP ability to strip and mirror data across a network that scales with the number of network participants. In conclusion, our software demonstrates that NFU-enabled IBP can operate as an effective framework for data storage and computation of biologically relevant algorithms provided that the algorithms can be converted to NFU-compatible formats (static shared C libraries). The greatest speedup would be in systems where the algorithms are amenable to parallelism. In addition to nucleotide FASTA alignments, suitable life science applications might include tools for genome-wide sequence data mining, like BLAST or other string matching algorithms, microarray data storage and analysis, and notoriously storage-demanding image generating technologies, including electropheragrams, flow cytometry, magnetic resonance imaging, and 2D gels. These results provide the foundation for further development of other distributed NFU-compatible software.

## Availability and requirements

▪ Project name: NFU-FASTA

▪ Project homepage: 

▪ Operating system(s): only tested with gnu compiler on Linux machines

▪ Programming language: C, Java

▪ Other requirements: IBP

▪ License: none

▪ Any restrictions to use by non-academics: none

## Abbreviations

DoS – Denial of Service

IBP – Internet Backplane Protocol

L-Bone – Logistical Backbone

LoRS – Logistical Runtime System

NFU – Network Functional Units

WAN – Wide Area Network

XML – eXtensible Markup Language

## Competing interests

The author(s) declare that they have no competing interests.

## Authors' contributions

EJB conceived of the study, participated in its design and coordination and drafted the manuscript. GNL created the Execution Upload Server and NFU-compatible FASTA source code, performed benchmark test cases, and participated in the creation of the manuscript. HL created the NFU extension for IBP. RK coded the DB Uploading Server and the XNDServer. All authors have read and approve the final manuscript.
